# Age-Related Alterations in SIBLING Proteins and Dentin Micro-Architecture: Morphological and Molecular Associations

**DOI:** 10.3390/life15121919

**Published:** 2025-12-15

**Authors:** Neshka Manchorova-Veleva, Mina Pencheva, David Baruh, Veselina Todorova, Lyubomir Vangelov, Margarita Guenova, Zhelyazko Damyanov, Donka Keskinova

**Affiliations:** 1 Department of Operative Dentistry and Endodontics, Faculty of Dental Medicine, Medical University of Plovdiv, 4002 Plovdiv, Bulgaria; veselina.todorova@mu-plovdiv.bg (V.T.); lyubomir.vangelov@mu-polvdiv.bg (L.V.); 2 Department of Medical Physics and Biophysics, Faculty of Pharmacy, Medical University of Plovdiv, 4002 Plovdiv, Bulgaria; 3 Department of Software Engineering, Faculty of Mathematics and Informatics, Sofia University “St. Kliment Ohridski”, 1164 Sofia, Bulgaria; dbaruh@uni-sofia.bg; 4 Laboratory of Haematopathology and Immunology, National Specialized Hospital for Haematological Diseases, 1756 Sofia, Bulgaria; margenova@gmail.com; 5 Central Laboratory of Mineralogy and Crystallography, Institute of Mineralogy and Crystallography, Bulgarian Academy of Sciences, 1113 Sofia, Bulgaria; damyanov@clmc.bas.bg; 6 Department of Applied and Institutional Sociology, Faculty of Philosophy and History, University of Plovdiv, 4000 Plovdiv, Bulgaria; d.keskinova@uni-plovdiv.bg

**Keywords:** age, dentin, DMP-1, DSPP, IHC, OPN, odontoblasts, PLM, SEM, SIBLING proteins

## Abstract

**Background**: Aging is associated with progressive structural and functional changes in dentin, reducing its mechanical integrity and increasing vulnerability to damage. Among the most important regulators of dentin physiology are extracellular matrix proteins from the SIBLING family, including Dentin Matrix Protein 1 (DMP-1), Dentin Sialophosphoprotein (DSPP), and Osteopontin (OPN). These proteins are essential for dentin mineralization, collagen organization, and tissue remodeling. Despite their critical role, knowledge about their age-related distribution and correlation with dentin structure and morphology remains limited. **Aim**: To examine age-dependent changes in the expression of SIBLING proteins (DMP-1, DSPP, OPN) in human dentin and to evaluate their relationship with collagen structure and ultramorphology using polarized light microscopy (PLM), immunohistochemistry (IHC), and scanning electron microscopy (SEM). **Materials and Methods**: Ninety extracted human teeth were categorized into young (≤17 years), mature (18–50 years), and old (>51 years) groups. IHC was applied to detect protein distribution, PLM to assess collagen organization, and SEM to evaluate dentinal morphology. **Results and Conclusions**: Aging was associated with increased expression of DMP-1 and OPN and a reduction in DSPP, which is particularly evident in peritubular dentin. Older samples showed collagen disorganization, reduced birefringence, and extensive intratubular mineralization. These findings suggest that age-related alterations in SIBLING proteins contribute to structural changes in dentin, providing new insights relevant to dental care in elderly patients.

## 1. Introduction

Dentin is a mineralized connective tissue that constitutes most of the tooth structure, ensuring biomechanical stability and participating in basic physiological processes such as load distribution and protection of dental pulp [1]. Unlike enamel, dentin remains biologically active throughout life, undergoing continuous remodeling and adaptive responses to mechanical, biochemical, and physiological stimuli [1,2].

Structurally, dentin is composed of a highly organized mineral phase, mostly hydroxyapatite crystals, embedded in a complex organic extracellular matrix (ECM) [3]. ECM is rich in type I collagen but also contains a variety of non-collagen proteins, phosphoproteins, and glycoproteins that play a critical role in regulating mineral deposition, tissue organization, and structural integrity [3,4,5].

Among these regulatory molecules, members of the SIBLING family (Small Integrin-Binding Ligand N-linked Glycoproteins) [6], including Dentin Matrix Protein-1 (DMP-1) [7], Dentin Sialophosphoprotein (DSPP) [8], and Osteopontin (OPN) [9], are recognized as key modulators of dentin biomineralization. DMP-1 is essential for both the early differentiation of odontoblasts and the subsequent organization of the mineralized matrix [7]. In the extracellular space, DMP-1 undergoes proteolytic processing, generating fragments that engage in hydroxyapatite nucleation and crystal growth regulation [7]. DSPP, which splits into DSP and DPP, is crucial for initiating the mineralization and structural stabilization of collagen fibrils [8]. OPN, a multifunctional glycoprotein, contributes to the control of mineral organization, especially matrix interfaces, and participates in cell adhesion, tissue repair, and immune modulation [9].

Age-related changes in dentin, often referred to as “dentin ageing,” encompass a wide range of structural, biochemical, and mechanical changes. These include increased mineral density, occlusion of dentinal tubules, disorganization of collagen fibers, and variations in ECM protein expression [10,11]. Such modifications can lead to increased tissue stiffness but reduced strength, which compromises the tooth’s resistance to mechanical stress and fractures, especially in the elderly [11].

Despite significant advances in understanding the composition and function of the components of the dentin ECM, data on how the expression and spatial distribution of SIBLING proteins change with age remain scarce. Furthermore, the relationship between these molecular changes and the structural and ultramorphological characteristics of dentin is not fully understood.

Advances in microscopic observation and testing methods provide new opportunities for the detailed study of dentin aging. Polarized light microscopy (PLM) allows visualization of the orientation and anisotropy of collagen fibers, serving as a marker of structural integrity [12]. In addition, scanning electron microscopy (SEM) provides a high-resolution image of the surface morphology and microstructure of dentin, allowing direct analysis of age-related changes in hydroxyapatite crystals and collagen matrix [13].

Despite in-depth studies of human dentin at the morphological, ultramicroscopic and molecular-biological levels, there is still a gap in knowledge regarding its comprehensive assessment in the process of physiological aging. There is a lack of convincing data on the molecular navigation of dynamic dentin mineralization as a natural response in the course of aging, and the non-collagen proteins in its composition as probable conductors in age-dependent dentinogenesis have not been sufficiently studied. In this regard, our study focused on a comparison between three proteins from the SIBLING family, and our specific hypothesis is that DMP-1 and OPN increase with age, while DSPP decreases. Against the backdrop of the ever-increasing life expectancy in the global population, such studies would bring additional knowledge about the protein-mineral architecture of human dentin in physiological aging and give a new direction in optimizing diagnostic and therapeutic methods in modern dental treatment.

The current study aims to analyze age-dependent changes in the distribution and expression levels of DMP-1, DSPP, and OPN in human dentin. In addition, the study aims to link these molecular findings to age-related changes in dentin structure observed by polarized light microscopy and SEM. Understanding these interrelationships is crucial for elucidating the mechanisms underlying dentine aging and is relevant for the development of age-specific preventive and restorative dental strategies.

## 2. Materials and Methods

### 2.1. Study Design and Sample Collection

This prospective study included a total of 90 human teeth, all of which were intact and extracted for clinical reasons. The teeth were removed due to periodontal indications (premolars and incisors), orthodontic reasons (developing third molars), or eruption difficulties (third molars).

The extracted teeth were categorized into three age-related groups based on pulp and dentin maturity:

- Group 1 (Developing pulp and dentin): Teeth from individuals aged up to 17 years (*n* = 30)
- Group 2 (Mature pulp and dentin): Teeth from individuals aged 18 to 50 years (*n* = 30)
- Group 3 (Aged pulp and dentin): Teeth from individuals older than 51 years (*n* = 30)

### 2.2. Inclusion and Exclusion Criteria

#### 2.2.1. Inclusion Criteria

- Intact human teeth extracted for clinical reasons
- Teeth extracted due to periodontal, orthodontic, or eruption-related indications
- Teeth from individuals within specified age groups (up to 17, 18–50, and older than 51 years)

#### 2.2.2. Exclusion Criteria

- Teeth with caries, restorations, or fractures
- Teeth with previous endodontic treatment
- Teeth with any pathological lesions (e.g., cysts, tumors).
- Teeth extracted due to trauma or non-clinical reasons.

### 2.3. Light Microscopy with Polarized Light Microscopy (PLM)

Following extraction, all teeth were stored in a 0.5% thymol solution at 4 °C for a maximum of 3 months to prevent protein degradation. From each tooth, both crown and root portions were sampled [14]. Two longitudinal or cross-sectional thin sections (~0.3 μm) were prepared per tooth and mounted on glass slides using Canadian balsam. In addition, two specimens from each tooth were embedded in epoxy resin and mechanically polished to a final thickness of approximately 50 μm to achieve a smooth observation surface.

Prior to embedding, all specimens were visually oriented in both longitudinal and transverse planes to ensure consistent sectioning relative to tooth anatomy.

Microscopic analysis was performed using a Leitz Orthoplan-Pol polarized light microscope, equipped with a digital imaging system. Three conventional polarized light microscopy modes were employed for the evaluation of crystalline anisotropic structures: bright-field mode; dark-field mode; polarization with a quartz compensator plate. This multimodal approach enabled detailed visualization of birefringent features and microstructural organization within the dental hard tissues.

### 2.4. Immunohistochemistry

The method describes an immunohistochemical study of paraffin-embedded pulpo-dentine tissue sections. After extraction, the teeth were fixed in 10% neutral formalin for 24 h and processed mechanically by trimming the enamel and the surface layer of dentin, to reduce the time for penetration of the decalcifying agent through the hard tissues and to preserve protein complexes in the pulp and dentin. These preparations were subsequently decalcified to ensure adequate antigen exposure for immunolabeling.

To determine an appropriate decalcification strategy for dentin, several protocols described in the bone biopsy literature were preliminarily tested on human dentin specimens, and antigen preservation was assessed by immunohistochemistry using SIBLING antibodies [15,16,17,18,19,20,21,22]. Based on these pilot comparisons, the protocol demonstrating the most reliable retention of SIBLING epitopes was selected for use in the present study.

According to the adopted protocol, the teeth were fixed in 10% formalin for 24 h, followed by enamel removal from the crown of the tooth and the outer half of the crown dentin in order to reduce the decalcification time, decalcification in 3% hydrochloric acid for 2 h, followed by a triple bath in distilled water for 15 min and repeated exposure to 3% hydrochloric acid for 2 h. Following decalcification, the samples underwent standard dehydration, clarification, and paraffin embedding [15].

Thin sections (2.0–2.5 μm) were processed through dewaxing, rehydration, antigen retrieval, and incubation with monoclonal antibodies to DMP-1 (LFMb-31, sc-73633), DSPP (LFMb-21, sc-73632), and OPN (AKm2A1, sc-21742), diluted 1:200 (Santa Cruz Biotechnology Inc., Dallas, TX, USA), using an automated Leica Bond-Max system (Leica Biosystems Ltd., Nussloch, Germany).

Optical microscopy and assessment of immune reactions were conducted using observation with a Leica DMI3000B light microscope and a camera for digital archiving of the observed specimens (DFC295; Leica Microsystems Ltd., Wetzlar, Germany).

Immunostaining intensity was evaluated using a semi-quantitative 3-grade scoring system (0 = no expression, 1 = weak expression, 2 = strong expression). All slides were scored independently by two blinded observers. Inter- and intra-observer agreement was assessed using Cohen’s kappa coefficient (κ), which showed substantial agreement (κ = 0.79–0.92). Discordant cases were reviewed together to establish a consensus score.

### 2.5. Scanning Electron Microscopy (SEM)

Fractured specimens were mounted on aluminum stubs using conductive carbon tape and sputter-coated with a thin (~10 nm) gold layer under vacuum (Polaron SC7640) to enhance surface conductivity. SEM analysis was performed using a Philips 515 SEM (Philips, Eindhoven, The Netherlands) at an accelerating voltage of 30 keV in high-vacuum mode.

SEM imaging was performed on a separate set of non-demineralized dentin specimens fixed in 10% neutral formalin for 6 h. The samples were split into two halves by controlled mechanical fragmentation, avoiding rotary instruments to prevent smear-layer formation. They then underwent standard three-step ethanol dehydration and vacuum sputter-coating with gold to preserve the mineralized dentin structure for morphological evaluation. These specimens were not exposed to hydrochloric acid, and a separate sample set was required because metallization and vacuum processing prevent reuse for FFDPE immunohistochemistry.

Imaging was conducted at magnifications ranging from ×500 to ×10,000 to evaluate dentin surface morphology, including dentinal tubule structure and peritubular/intertubular organization. Representative micrographs were digitally acquired under standardized conditions for comparative and quantitative analysis using ImageJ 1.53c software (NIH, Bethesda, MD, USA).

### 2.6. Statistical Analysis

Descriptive statistics were applied to summarize the distribution of variables, including the expression patterns of DMP-1, DSPP, and OPN, as well as age, sex, and topographic characteristics of the dentin samples. Non-parametric tests were used due to the ordinal nature or non-normal distribution of the data.

The Kruskal–Wallis test was employed to assess differences between more than two independent groups, while the Mann–Whitney *U* test was used for pairwise comparisons. These analyses were applied to both dependent and independent variables with more than two levels, such as regional expression patterns or stratification by age groups, and a *p*-value of less than 0.05 was considered statistically significant.

## 3. Results

### 3.1. Observations by Light Microscopy with Polarized Light Microscopy

Polarized light microscopy is a reliable method for studying dentine structure, allowing visualization of the integrated organic and inorganic components without destroying the mineral phase. This approach reveals details about crystalline morphology, crystalline architectonics, phase heterogeneity and optical properties of different dentine layers in different age groups, as presented in Table 1.

The main structural features of dentin in healthy areas described in the literature are consistent with our observations, but systematic studies of age-dependent changes, especially in crown and root dentin, remain limited. The current data highlight that tracking these changes by polarization microscopy provides valuable information about the age-related dynamics of dentinal organization and justifies the need for further research.

**Figure 1:**
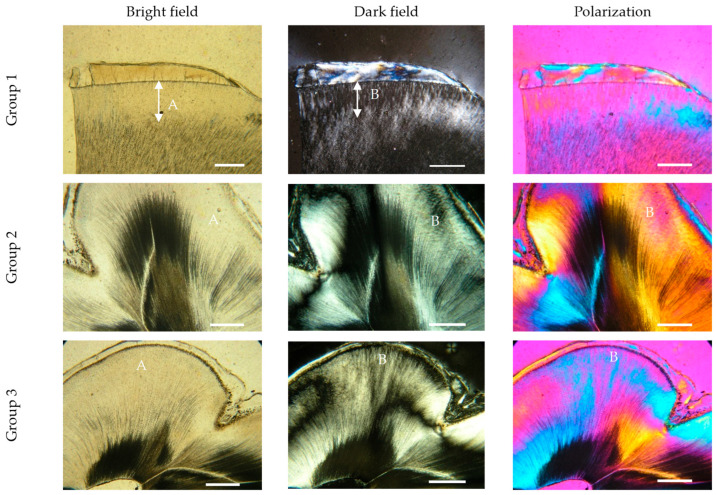
Photomicrographs with transmitted light microscopy of crown dentin in the three age groups, with linear polarization in light, dark field mode and polarization with a quartz plate. Group 1: (A) atubular dentin light field (arrows); (B) phase characteristic, with heterogeneous apatite organization observed in a dark field (arrows); Group 2: (A) wider areas of atubular dentin in a bright field; (B) dark field and polarization reveals inhomogeneous apatite-protein architectonics; Group 3: (A) zones with narrowing and complete obliteration of dentinal tubules, which are very wide and are localized not only under the cementodentin junction (CDJ); (B) the phase differences in the individual dentinal words are deepened, with areas of complete tubular sclerosis predominating. Molar specimen. Scale bars = 0.3 mm.

In group 1 (young people), dentin is predominantly tubular, with atubular dentin being limited to the area immediately below the enamel-dentin border, forming the mantle layer. Under a light and polarization microscope, the structural heterogeneity of these zones stands out—especially near the pulp, where the granular texture and color differences under polarization show variations in the crystalline organization of the hydroxyapatite and apatite-protein matrix. The tubes are wide and open, and the atubular dentin remains stable and dense (Figure 1, group 1).

As we age (group 2), the atubular dentin expands and now encompasses the circumpulpal dentin. Under a dark field, a characteristic granular texture of these zones is noticeable, and quartz plate polarization reveals color differences that reflect an inhomogeneous distribution of mineral components and apatite-protein architecture. Some tubules begin to narrow or become partially obliterated, but there are no signs of transparent sclerotic dentin (physiologically reactive dentin). The dentin gradually loses its homogeneity, as the atubular zones grow and acquire different optical characteristics (Figure 1, group 2).

In teeth over 51 years of age (group 3), the atubular dentin extends over the entire thickness of the crown dentin, and the areas below the enamel-dentin border are no longer the only atubular areas. The dentinal tubules are significantly narrowed or completely obliterated, and the granular structure and phase changes under a dark field reflect the natural aging processes. Polarization highlights differences in crystal organization and apatite-protein architecture, with color variations showing different mineral characteristics of the atubular and tubular zones. These changes are not the result of reactive dentin against bacterial invasion but indicate the gradual aging and sclerosis of the dentin, which leads to reduced homogeneity and alteration of the optical and structural properties of the tissue. These physiological changes in dentin with age are defined as transparent sclerotic dentin or physiologically reactive dentin, in contrast to the protective forms in tertiary dentinogenesis (Figure 1, group 3).

**Figure 2:**
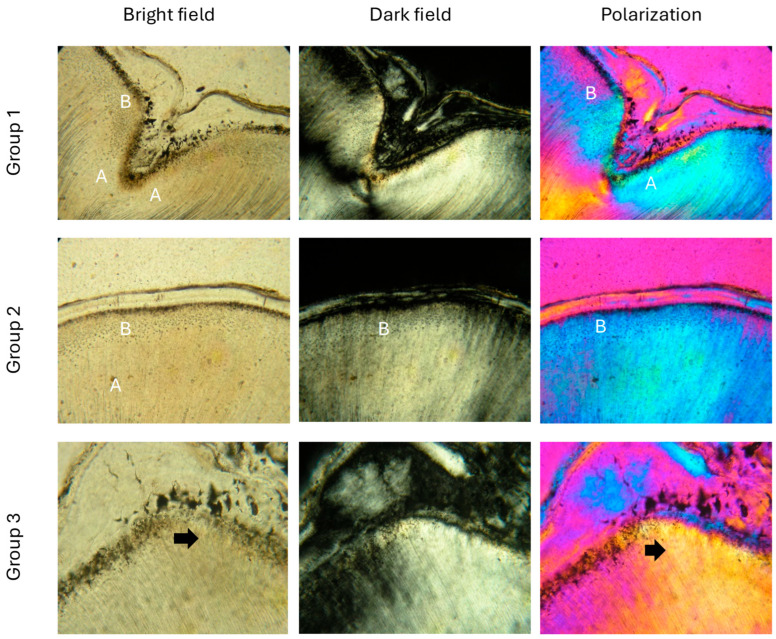
Photomicrographs with transmitted light microscopy of root dentin, with linear polarization in the mode of light, dark field and with a quartz plate. Group 1 and group 2: (A) atubular spaces are observed; (B) granular layer of Tomes immediately below the CDJ; Group 3: (black arrow) the zones of narrowed and sclerosed tubules are very large, larger interglobular spaces are visible, with an enlarged globular layer; Molar specimen. Scale bars = 0.3 mm.

In young people (group 1 (<17 years)), root dentin near the cementodentin junction (CDJ) shows a characteristic granular layer. The atubular zones are wider than those in the crown dentin of the same age. The tubules are open, and the area of the granular layer of Tomes is clearly visible immediately below the CDJ. Under polarized light, the structural heterogeneity of the dentinal matrix stands out, with the interglobular spaces remaining small and uniform (Figure 2, group 1).

In group 2, the atubular spaces in the root dentin become even wider, and structural heterogeneity increases. Under polarized light, more pronounced variations occur that reflect changes in mineral composition. The tubules begin to narrow and the interglobular spaces grow. The granular layer of Tomes is still visible, but gradually changes with age (Figure 2, group 2).

In group 3 (age over 51 years and especially after 65 years), the atubular zones cover almost the entire root dentin. The tubules are massively sclerosed and narrowed, and the interglobular spaces are significantly enlarged. Polarization microscopy shows a change in the mineral structure of dentin, including an enlarged globular layer. Dentin is more heterogeneous, with impaired apatite-protein organization, which reflects tissue aging (Figure 2, group 3).

### 3.2. Results Obtained After Immunohistochemical Staining

The immunohistochemical method used showed that the distribution of DMP-1, DSPP and OPN markers in odontoblasts and dentin tissues showed variability associated with age-related changes and specific areas of mineralization. Their expression was weaker in the dentin and pulp of dental germs and increased significantly from mature to old pulpo-dentin samples, which emphasized their role in mineralization and maintenance of dentin structure.

**Figure 3:**
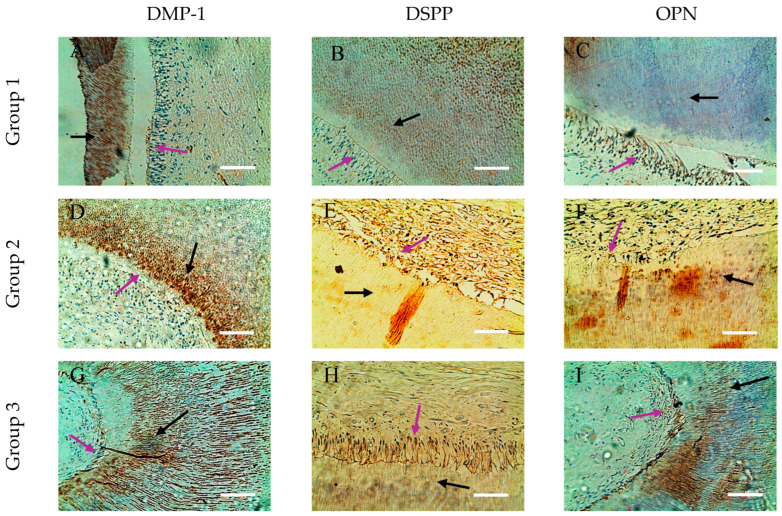
Comparative distribution in dentin and odontoblasts of SIBLING proteins in the crown of dental specimens. (**A**,**D**,**G**) Expression of DMP-1 in circumpulpal dentin (black arrow) and in odontoblastic cells (purple arrow); (**B**,**E**,**H**) expression of DSPP in odontoblasts (purple arrow) and immunopositive reaction in circumpulpal dentin (black arrow); (**C**,**F**,**I**) OPN is well represented in odontoblasts (purple arrow), with a spotted appearance in dentin (black arrow); ×200. Scale bars = 0.3 mm.

**Figure 4:**
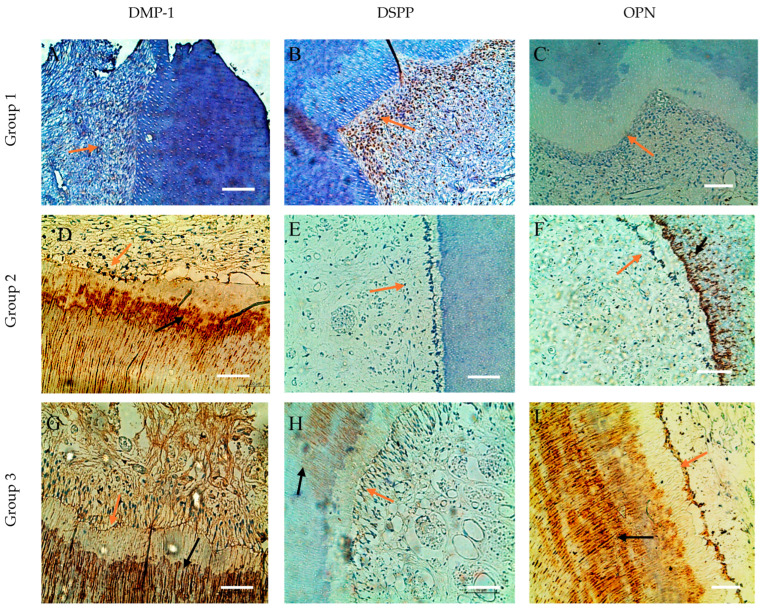
Comparative distribution in dentin and odontoblasts of SIBLING (DMP-1, DSPP, OPN) proteins from the root of dental specimens. (**A**) Weak expression of DMP-1; (**B**) increased expression for DSPP in odontoblasts; (**C**) weak expression for OPN in odontoblasts (orange arrow); (**D**–**F**) expression of DMP-1, DSPP, OPN in odontoblasts (orange arrow) and, respectively, increased expression in dentin for DMP-1 and OPN (black arrow); (**G**–**I**) Increased expression in dentin for DMP-1, DSPP, OPN (black arrow) and absence expression for DSPP (orange arrow); ×200. Scale bars = 0.3 mm.

Immunohistochemical analysis of SIBLING proteins (DMP-1, DSPP, OPN) in the crown and root of dental specimens confirms zonal differences in expression, consistent with the molecular analysis. Specifically, OPN was decreased and DSPP was increased in the apical segments (Figure 3 and Figure 4).

In the group 1 dental samples, a specific distribution of the three SIBLING proteins in the dentin and odontoblasts was observed. DMP-1 is visualized primarily in the deep layers of the circumpulpal dentin, forming ring-like structures clearly separated from the odontoblastic palisade and the immunonegative predentin. Furthermore, DMP-1 is concentrated in the metadentin zones, mineralization front, and dentin tubule walls, while intertubular dentin remains immunonegative (Figure 3A and Figure 4A). For DSPP, we found expression mainly in odontoblasts, which form a linked network through intercellular contacts and pulpo-dentin membrane; the circumpulpal dentin is weakly positive, as its distribution in the peritubular dentin is uneven, and the intertubular dentin remains immunonegative. Interestingly, in the same sample, some odontoblasts are DSPP-positive, and others are DSPP-negative, which highlights the heterogeneity of expression in cells (Figure 3B and Figure 4B).

For OPN, we also reported expression in odontoblasts, with its distribution in dentinal tissue being uneven, mottled and inhomogeneous, with marker-rich areas and immunonegative sites, suggesting dynamic localization and potential involvement in different stages of mineralization (Figure 3C and Figure 4C).

In group 2, expansion and enhancement of DMP-1 expression in the circumpulpal dentin was observed, where the walls of the dentinal tubules were clearly marked, while the predentin remained immunonegative (Figure 3D and Figure 4D). DSPP is expressed mainly in odontoblasts, but dentin remains weakly immunopositive (Figure 3F and Figure 4F). OPN shows a DMP-1-like distribution in the circumpulpal dentin, with its inhomogeneity and spotty-like organization being preserved, although some of the dentinal tissue remains negative (Figure 3F and Figure 4F). Odontoblasts in mature pulp are positive for all three markers, emphasizing the preservation of their functional activity in the process of mineralization with age.

In the aged specimens (group 3), DMP-1 and OPN continue to demonstrate wide distribution and high expression in the circumpulpal dentin, with DMP-1 concentrated in calculospherites—zones of active mineralization (Figure 3G and Figure 4G). OPN retains its inhomogeneous spot-like distribution, including striated areas (Figure 3I and Figure 4I), while DSPP remains mainly limited to individual odontoblastic cells and small dentin sites, with dentin being largely immunonegative (Figure 3H and Figure 4H). Odontoblasts in old pulp exhibit expression of all three markers, albeit DSPP in limited areas, indicating that as we age, DMP-1 and OPN play a major role in maintaining dentin mineralization activity, while DSPP probably loses its direct functional role.

We can conclude that DMP-1 exhibits a consistent ring-like and highly expressive distribution in the deep layers of dentin and in odontoblasts across all age groups, highlighting its key role in mineralization. DSPP is localized primarily in odontoblasts and in limited dentinal areas, and its expression decreases with age. OPN shows a more uneven, spotted-like distribution, with an active presence in odontoblasts and partially in the circumpulpal dentin, and its role appears to be complementary to DMP-1 in mineralization processes. Predentin remains immunonegative for all three markers, regardless of age, which emphasizes its different functional state compared to mineralized dentin.

### 3.3. Statistical Analysis of Immunohistochemical Assessment Data

The results in Table 2 show that the markers studied—DMP-1, DSPP and OPN—vary in their expression according to age and location. DMP-1 demonstrated high expression (grade 2) in odontoblasts of all age groups, while in dentin expression increased with age and reached statistically significant values in older individuals. DSPP is strongly expressed in odontoblasts (75.9%—grade 2), but low expression predominates in dentin (grade 0—75.9%), especially in mature and old samples. OPN shows high expression in odontoblasts, which increases with age; in dentin, a sharp increase in expression was reported in adult patients (75%—grade 2).

With age, there is a significant increase in positive immunolabeling of DMP-1 markers (in both methods of assessment) and OPN/D, with the lowest values in young individuals and significantly higher values in mature and aged individuals. For DSPP and OPN/ODS, there are no statistically significant differences between age groups. This indicates that DMP-1 and OPN may have a more active role in mature and old tissues (Table 3).

The results in Table 4 show that most markers have no statistically significant differences between the sexes (*p* > 0.05). Only OPN/Ods showed a significant difference (*p* = 0.020), with women having a higher proportion of strong expression (90.7%) compared to men (70.0%). This suggests a sex difference in OPN expression.

Our results show that in women, positive immunolabeling for OPN in odontoblasts is statistically greater compared to men. A possible explanation can be sought in interregulatory processes with steroid hormones (Table 4).

The results of Table 5 show that there are no significant differences between masticatory and frontal teeth in most markers (*p* > 0.05). Only OPN/D showed a statistically significant difference (*p* < 0.01): frontal teeth had significantly higher expression (score 2—89.5%) compared to masticatory teeth. In our material, OPN immunolabeling was more pronounced in anterior dentin (*p* < 0.01), which may be relevant to regional differences in age-related dentin characteristics.

In terms of age, there is a clear trend towards increasing expression with age, especially for DMP-1 and OPN.

For DMP-1/Ods and DMP-1/D, we measured the lowest expression in young pulp specimens and the highest in old pulp specimens, while the expression of DSPP/Ods was consistently high at all ages. On the other hand, DSPP/D is more pronounced in young individuals, and OPN/Ods and especially OPN/D increase significantly with age, with the strongest expression reported in samples of old pulp and dentin. The results showed that age had a significant impact on the expression of some markers, such as DMP-1 and OPN, while localization was mainly relevant for OPN/D.

The data from Table 6 report the expression of DMP-1/Ods in the lower teeth and show more often low expression (score 0—21.1%) compared to the upper teeth (2.2%), and this difference is statistically significant (*p* < 0.05). High expression prevails in the upper teeth (score 2—71.1%). For DMP-1/D, high expression prevails in both types of teeth (score 2—over 75%). There are almost no cases of low expression in upper teeth. DSPP/Ods is characterized by high expression in both the upper and lower teeth, with the lower teeth having a slightly higher proportion (Table 6 (81.6%)).

DSPP/D is dominated by low expression (score 0—about 76%) in both types of teeth. Moderate expression (grade 1) is more common in the upper teeth (15.6% versus 2.6% in the lower teeth). In OPN/Ods, high expression is more common in upper teeth (86.7%), while lower teeth are more common in low scores (0 and 1). OPN/D showed relatively balanced expression, with a high score (2) being slightly more common in upper teeth (60%) versus lower teeth (52.6%) (Table 6).

### 3.4. Scanning Electron Microscopy (SEM) Observations

The study of the ultrastructure of root dentin by scanning electron microscopy (SEM) reveals key features contributing to a better understanding of the morphology and composition of dentin tissue. A network-like structure has been observed in the peritubular dentin, possibly composed of non-collagen proteins from the SIBLING and SLRP families, confirming the existence of significant differences from intertubular dentin. Despite the lack of specific enzymatic processing, fixation with neutral formalin likely led to partial degradation of protein-associated macromolecules, allowing the visualization of structures inaccessible in conventional SEM approaches.

**Figure 5:**
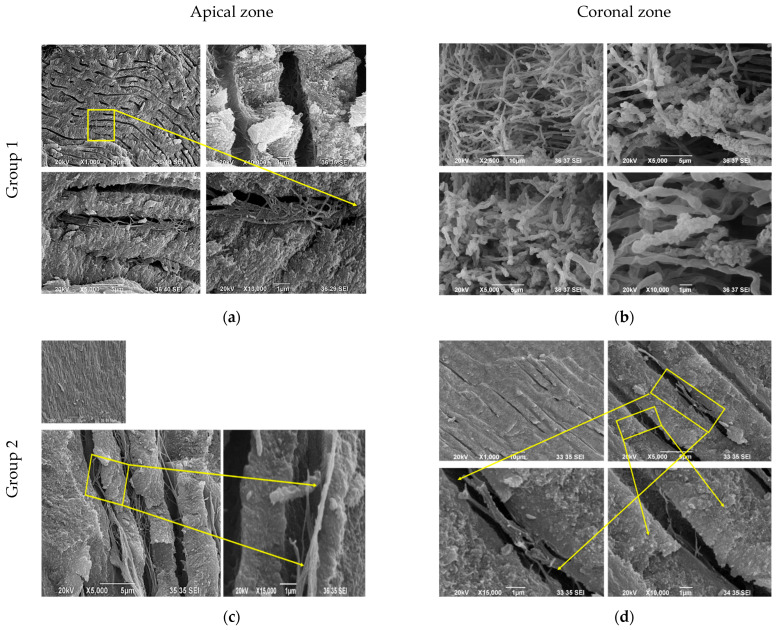

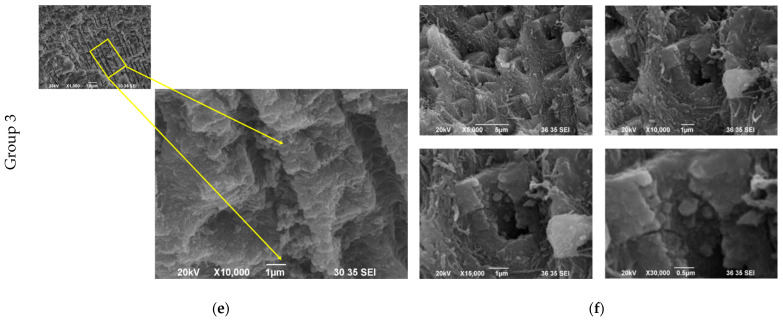
Photomicrographs from SEM of coronal and apical root dentin. (**a**) There is a lamina limitans, which separates the intertubular from the peritubular dentin; (**b**) Fibrillary structures (collagen and non-collagen) integrated with crystallites shall be observed; (**c**) Semi-concentric and prismatic-like radial fibrillar units of the peritubular dentin; (**d**) Fibrillary structures of the peritubular dentin are illustrated; (**e**) Intratubular mineralization, confluent crystalline grains, formation of larger grape-like conglomerates are observed; (**f**) dense mineral structure and a deposit of crystals are visible intratubularly; ×500; ×1000; ×1500; ×2000; ×2500; ×5000; ×10,000.

SEM photomicrographs of young root dentin taken from the apical zone show characteristic morphology of dentin structure at different magnifications. A lamina limitans is clearly visualized, separating the intertubular dentin from the peritubular dentin, and outlining a distinct difference in microstructure between the two dentin types (Figure 5a).

Intertubular dentin exhibits a fine, three-dimensional collagen network located between the dentin tubules. In most of the tubules, processes of odontoblast cells are observed, which is characteristic of young dentin and indicates active cellular function. The coronary dentin of young teeth is distinguished by a typical tubular structure, with the diameter of the tubules varying, despite their uniform arrangement in one transverse plane. Areas without tubules are also noted, and the distribution of tubules is heterogeneous. At high magnification (up to ×10,000), fibrillar structures, possibly collagen or non-collagen, integrated with mineral crystallites are found (Figure 5b).

The peritubular dentin is well formed, with a characteristic annular hypermineralized structure, clearly distinguished from the intertubular dentin.

In the young root dentin in the apical zone, at different magnifications, the lamina limitans are clearly revealed, which separates the intertubular from the peritubular dentin, highlighting the morphological differences between the two types. Intertubular dentin is characterized by a fine collagen network located between the dentin tubules, with growths of odontoblastic cells observed in numerous tubules.

The lamina limitans is an electron-dense, canvas-like structure that separates the peritubular from the intertubular dentin [15]. It contains proteoglycans, the main structural component being the skeletal protein (core protein) associated with glycosaminoglycans (GAG) [23]. The lamina limitans forms a thin sheath around the dentin tubules and plays a key role in the organization of peritubular dentin.

SEM analysis of mature root dentin in the coronary zone (Figure 5c,d) illustrates fibrillar structures originating from the peritubular dentin. With age, progressive intratubular mineralization is observed, leading to partial or complete calcification of the tubules, known as tubular sclerosis. Although the structures described above (collagen network, peritubular zone, fibrillary inclusions) are still observed in mature root dentin, they are more limited, less pronounced and often accompanied by structural changes such as confluent mineral deposits, loss of the annular mineralized border and narrowing of the tubular lumen.

SEM images of old root dentin in the apical zone (Figure 5e,f) demonstrate significant intratubular mineralization, with the formation of confluent crystalline grains and larger grape-like conglomerates. Tubular sclerosis is pronounced, with a visible narrowing of the intratubular space and the absence of the characteristic hypermineralized annular zone around the dentinal tubules. These changes are especially pronounced in the apical part of the root, where mineral deposits are more abundant compared to the coronary zone. There is a loss of clearly identifiable peritubular dentin with age.

**Figure 6:**
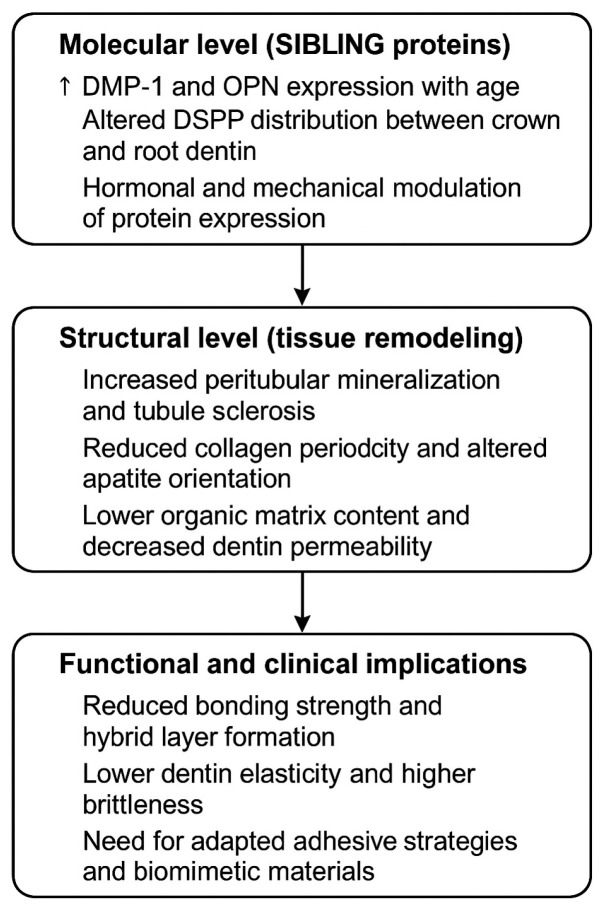
Model of age-related dentin remodeling. (**↑**) Arrow indicate progression.

In Figure 6 is presented a model summarizes the proposed relationship between SIBLING protein expression, age-related dentin remodeling, and its functional and clinical implications.

## 4. Discussion

In the course of the study, light and scanning electron microscopy revealed a number of interesting and multi-layered changes in the structure and optical characteristics of dentin tissue in different age groups. The combined use of light field, dark field, and quartz plate polarization allowed us to make a comprehensive overview of structural changes in dentin. Thanks to the ability of polarization microscopy to visualize birefringent structures, we observed morphological and phase differences in the apatite-protein architecture of the tissue.

In the specimens of young/developing human teeth, sections of coronal and apical root dentin show clearly differentiated tubular and atubular zones, with the latter having a homogeneous appearance in a light field and a granular structure in a dark field. Quartz plate polarization microscopy highlights color differences reflecting variations in crystalline organization. With age, the atubular zones expand and become more diverse, exhibiting internal inhomogeneity and different crystalline orientations. In patients over 51 years of age, these changes are especially pronounced—the tubules are almost completely obliterated, and the structure acquires a vitreous character, accompanied by distinct color transitions, indicating age-related changes in the crystalline architecture of the dentin [12,13].

On the other hand, SEM allowed us to reveal ultramorphological details that often remain hidden under standard observation methods. In the young crown dentin, we observed an extremely complex fibrillar architecture, in which collagen and non-collagen proteins built a lattice that “locked” the apatite crystals. This arrangement gives the impression of a dynamic but well-organized structure, which has also been observed in earlier studies [24].

Our observations showed that with age, there is a clear reorganization of the dentin structure, expressed mostly as increased mineralization and compaction of the peritubular dentin, which correlates with the onset of sclerosis of the tubules. These results prove the hypothesis of physiological sclerosis of dentin proposed by Garcés-Ortíz et al. (2015) [25]. Also, they are consistent with earlier observations that describe the age-related increase in intratubular mineralization as a defense mechanism against caries and mechanical wear [24,25]. On the other hand, Kabartai et al. (2015) focus on the dynamics of the mineral phase and its influence on the elasticity and strength of dentinal tissue, which supports our conclusions on the functional significance of age-related changes [26].

One of the most interesting findings of our study is the discovery of a network-like structure in root dentin, which SEM shows to be different from the classical collagen organization. The lack of the characteristic D-periodicity of collagen in this area suggests a significant role of non-collagen proteins, especially representatives of the SIBLING family—DMP-1, DSPP and OPN. These proteins are well known for their regulatory functions in mineralization and their ability to modulate the growth of apatite crystals [27,28]. Our results support the model according to which SIBLING proteins form the organic matrix that participates in both the spatial organization of the mineral phase and its morphology.

The current study provides new data on the expression of the SIBLING proteins DMP-1, DSPP and OPN in human dental dentin and odontoblasts, highlighting their age, sex and anatomical profiles. The results obtained support and complement earlier observations in the field [27,28,29,30].

In addition, our findings indicate that the structural heterogeneity of root dentin could be directly related to variations in the expression and localization of these SIBLING proteins [31]. Such variations may underlie the adaptive capacity of dentin to mechanical stress and age-related changes in mineralization [32]. The observed network-like organization might represent a specialized microenvironment that facilitates ion exchange and crystal nucleation, thus ensuring the balance between stability and remodeling within the dentin matrix [33,34]. Furthermore, the involvement of DMP-1, DSPP, and OPN in these processes suggests a coordinated regulatory mechanism, where each protein contributes distinct yet complementary functions [35]. DMP-1 is likely to act as a nucleator and spatial organizer for hydroxyapatite deposition, while DSPP, through its cleavage products DSP and DPP, may modulate crystal size and orientation [36]. OPN, on the other hand, is known for its inhibitory control over crystal growth, preventing uncontrolled mineral aggregation [37]. The interplay between these molecules could explain the fine-tuned mineral architecture observed in mature root dentin [38]. Taken together, the evidence points toward a more dynamic and complex organization of the dentin matrix than previously assumed. Rather than a static collagen-dominated scaffold, the root dentin appears to be a composite system, where non-collagenous proteins play a decisive role in defining both ultrastructural arrangement and functional properties [39]. These insights open new perspectives for understanding dentin pathophysiology and could have implications for the development of biomimetic materials aimed at dentin repair and regeneration [40].

As shown by George et al. (2008), age-related changes in DMP-1 expression are associated with increased remodeling activity in dentin [28]. Our data also showed a significant increase in DMP-1 and OPN expression with age, which fits the compensatory remodeling model in dental tissue [28,29,30]. Various studies have noted sex differences in the regulation of OPN in bone tissue, which are likely due to hormonal influences [30,41]. Estrogens, for example, have shown the ability to modulate the transcription of the gene for OPN through estrogen-receptor-mediated pathways, resulting in changes in mineralization and remodeling processes in bone [42]. Given the proximity in composition and remodeling mechanisms of bone and dentin tissue, it is logical to assume that similar hormonally determined variations can also exist in the teeth. Our finding of higher expression of OPN in odontoblasts in women supports this hypothesis and highlights the potential role of sex hormones in dentin matrix regulation [41,43,44,45,46].

Interestingly, there is a difference in OPN expression between frontal and masticatory teeth that has not been reported in detail in previous studies [47,48]. This finding suggests that functional load and morphological features may modulate protein expression in the dentin matrix. This hypothesis is consistent with observations of regional variations in dental dentin remodeling [48,49,50].

Zonal expression analysis revealed a significant decrease in OPN in the apical segments, while DSPP showed increased expression in the same area. The contrasting behavior of SIBLING proteins supports the idea of differentiated protein regulation between the crown and root parts of the tooth, indicating functional separation in dentinal biology [48,51].

In our study, higher OPN expression was observed in female samples compared to male samples. This finding may have clinical relevance, as OPN is known to be a hormone-responsive phosphoprotein involved in mineralization dynamics and extracellular matrix remodeling. Several studies have demonstrated that estrogen signaling can upregulate OPN transcription through estrogen receptor-mediated pathways, influencing mineral deposition rates and tissue turnover in mineralized tissues [52,53,54,55].

Experimental models also indicate that reduced estrogen levels are associated with alterations in OPN expression and changes in the mechanical properties of dentin and bone [56].

Given the compositional and developmental similarity between bone and dentin, it is plausible that sex hormone–dependent regulation of OPN may contribute to subtle structural and functional differences in dentin between males and females, particularly with aging [57,58]. These variations could potentially influence clinical outcomes such as bonding performance, dentin sensitivity, and long-term stability of restorations in older individuals [59].

However, the present study was not designed to directly evaluate clinical effects, and therefore these implications should be interpreted cautiously. Further studies integrating hormonal profiling, functional testing, and clinical restorative performance are required to clarify the impact of sex-related OPN differences on dentin biology.

Lower expression of DMP-1 in the lower teeth may reflect local differences in mechanical stress and blood supply, consistent with the observed heterogeneity in tooth structure and function according to their location. Studies on dental pulp have shown that it is a highly blood-supplied tissue, suggesting that blood supply may affect DMP-1 expression. Studies on dental pulp stem cells have shown that mechanical stimuli, such as tension and blood supply, can affect the differentiation and mineralization of these cells, suggesting that local mechanical conditions may affect DMP-1 expression [60].

The regulation of SIBLING proteins during aging is known to involve several molecular pathways related to mineral metabolism and extracellular matrix turnover. DMP-1 and OPN, for example, are regulated through signaling cascades involving PHEX and MEPE, which modulate phosphate homeostasis and mineral deposition balance [61,62].

Additionally, OPN interacts with integrin receptors, including αvβ3, activating downstream pathways such as MAPK and NF-κB, which contribute to remodeling and cell–matrix communication [63,64]. DSPP processing is influenced by matrix metalloproteinases (MMPs), particularly MMP-2 and MMP-20, which cleave DSPP into functional fragments that directly participate in dentin mineralization [65,66]. Age-related changes in MMP activity and shifts in phosphate-regulating pathways may therefore underline the observed alterations in SIBLING protein distribution in aged dentin [67,68]. These molecular interactions support the concept that SIBLING protein expression changes reflect adaptive remodeling processes rather than passive degradation [69].

In this context, data on the expression of SIBLING proteins indicate that they are not uniformly distributed in dentinal tissue, but have a specific localization associated with tubule topography and phase transitions in mineralization [42]. This finding is consistent with the reports of Gezawi et al. (2019), which highlight the role of DMP-1 and DSPP in initiating mineral deposition, while OPN is associated with the regulation of mineral density and stability [41]. In the same study, it was found that the phosphorylated forms of DSPP and DMP-1 can act as crystallization nuclei for the formation of apatite crystals in the presence of collagen, with DSPP inducing highly organized mineralization of collagen fibers and DMP-1 stimulating the deposition of mineral particles along their axis [43].

These molecular-level alterations in SIBLING protein distribution and processing during aging may also translate into clinically relevant changes in dentin structure and behavior. From a clinical perspective, the age-related modifications in SIBLING protein expression may contribute to changes in dentin mineral density, tubule sclerosis, and collagen integrity, which are all factors known to reduce bonding effectiveness in older dentin.

Studies have shown that aged dentin typically exhibits reduced hybrid layer formation and decreased resin infiltration due to lower porosity and altered organic matrix reactivity [70]. The increase in DMP-1 and OPN expression may represent a compensatory remodeling response that enhances mineral deposition, leading to a more sclerotic and less permeable substrate, while decreased DSPP may contribute to reduced elasticity and increased brittleness [71]. These biochemical changes help explain clinical observations of lower adhesive performance and decreased long-term durability of restorations in elderly patients [72,73]. Recognizing these molecular and structural shifts supports the use of modified bonding strategies in older dentin, including longer etching times, selective demineralization approaches, biomimetic remineralization primers, and MMP inhibitors to stabilize the hybrid layer [74,75].

The integration of morphological and molecular data allows for a deeper understanding of the biophysical processes taking place in dentin tissue with age and in different areas of the tooth. This is key not only for basic science, but also for clinical practice, as understanding the processes of mineralization and sclerosis can lead to the development of new strategies for the prevention and treatment of mineralized dental tissues.

The findings of this study provide new insight into dentin aging by demonstrating zone-specific structural remodeling and a sex-related difference in OPN expression. These results indicate that dentin aging is an active, regulated process rather than passive mineral accumulation. The coordinated changes in DMP-1, DSPP/DPP, and OPN expression suggest that SIBLING proteins function as key modulators of mineral deposition and collagen–apatite organization throughout life. Recognizing these molecular–structural relationships may support the development of biomimetic and regenerative strategies for improving dentin quality and restorative outcomes.

Future research should investigate the functional interactions between SIBLING proteins and the mineral phase of dentin, as well as the influence of environmental, nutritional, and systemic health conditions on dentin structure and regulation. Clarifying how individual SIBLING proteins contribute to age-related dentin changes may support the development of biomimetic and regenerative approaches aimed at improving tissue quality and restorative outcomes. Additionally, comparative studies of adhesive performance and clinical success in young versus aged dentin, and across different tooth regions or sex groups, are essential for translating these findings into evidence-based restorative and preventive strategies.

This study has limitations. Quantitative analysis of SEM data was not performed, which restricts direct evaluation of mineralization patterns. Donor-related systemic factors were not controlled, which may have influenced the observed protein expression. Immunohistochemistry was conducted on decalcified samples, allowing spatial localization but not precise quantification; thus, a semi-quantitative scoring system was used. Future studies should incorporate non-demineralized specimens and controlled clinical variables to strengthen the correlation between molecular expression patterns and structural properties.

## 5. Conclusions

The combination of polarization light microscopy, immunohistochemistry and SEM made it possible not only for morphological description but also for the detection of structural and phase changes in dentin tissue. The analysis of the data proved that with age, sclerotic changes by thickening of tubular walls dominate in the crown, while in the root portion, intratubular granular deposits and the role of lamina limitans are emphasized. Particularly significant is the identification of a network-like organization in peritubular dentin, different from the classical collagen architecture of intertubular zones, in which SIBLING proteins (DMP-1, DSPP, OPN) probably play a key role in the mineralization and modulation of apatite crystals. This discovery adds a new perspective to the understanding of dynamics in organic-mineral interactions in dentin, particularly the non-collagenous nature of peritubular areas as a new mechanism of formation and mineralization of dentin in aging. It highlights the transformation of tissue from a porous and elastic to a harder but more brittle structure. Such changes have direct clinical implications for restorative and endodontic practice and open the potential for future biomimetic research.

## Figures and Tables

**Table 1:** The main microscopic differences between developing, matured and aged dentin in different observation regimens.

Regime/Age	Developing and Matured Dentin	Aged Dentin
Bright field	The tubules are wide, the areas of sclerosis are small; interglobular spaces—small, less common	The areas with narrowed and sclerosed tubules are extensive; interglobular spaces—larger and more frequent
Dark Field	Sclerotic zones—few and with little reflection; interglobular spaces—small dark areas	Sclerotic zones—large, highly reflective; interglobular spaces—distinct dark “spots”
With quartz plate (polarized light)	Normal dentin—uniform interference colors (yellow-blue or orange-blue); sclerotic zones—few and similar in color	Sclerosed dentin—differently colored birefringence (paler, violet or almost colorless areas); interglobular spaces—without birefringence (black)

**Table 2:** Distribution of DMP-1, DSPP, and OPN expression across ages.

Marker	Score	Young (A) %	Mature (B) %	Old (C) %	Total %
DMP-1/Ods	0	55.6 B C	2.6	8.3	10.8
	1	11.1	28.9	19.4	22.9
	2	33.3	68.4	72.2	66.3
DMP-1/D	0	33.3 C	0.0	5.6	6.0
	1	22.2	10.5	11.1	12.0
	2	44.4	89.5 A	83.3 A	81.9
DSPP/Ods	0	11.1	10.5	5.6	8.4
	1	0.0	18.4	16.7	15.7
	2	88.9	71.1	77.8	75.9
DSPP/D	0	55.6	86.8	69.4	75.9
	1	11.1	10.5	8.3	9.6
	2	33.3 B	2.6	22.2 B	14.5
OPN/Ods	0	22.2	7.9	5.6	8.4
	1	11.1	13.2	8.3	10.8
	2	66.7	78.9	86.1	80.7
OPN/D	0	77.8 C	36.8	16.7	32.5
	1	11.1	13.2	8.3	10.8
	2	11.1	50.0	75.0 A	56.6

Three age groups: developing/young (A), mature (B), and aged (C) indicate statistically significant differences (e.g., A = *p* < 0.01 vs. group B); Expression scored (0—no expression, 1—weak, 2—strong expression); Markers: DMP-1, DSPP, OPN; Odontoblasts (Ods), dentin (D) samples.

**Table 3:** Distribution and expression of biomarkers in dentin after secondary grouping by immunoreactivity (negative/positive) and age-related changes.

Marker	Mean Developing (A)	Mean Mature (B)	Mean Aged (C)	*p*-Value
DMP-1/Ods	0.44	0.97 A	0.92 A	*p* = 0.000
DMP-1/D	0.67	1.00 A	0.94 A	*p* = 0.001
DSPP/Ods	0.89	0.89	0.94	*p* = 0.713
DSPP/D	0.44	0.13	0.31	*p* = 0.071
OPN/Ods	0.78	0.92	0.94	*p* = 0.275
OPN/D	0.22	0.63 A	0.83 A	*p* = 0.002

Note: Groups: Developing (young) (A), mature (B), and aged (C); Markers analyzed: DMP-1, DSPP, and OPN in dentin (D) and odontoblasts (Ods) samples; *p* < 0.05.

**Table 4:** Comparison of biomarker expression between female and male groups.

Marker	Score	Female n	Female % (A)	Male n	Male % (B)	Total n	Total %	*p*-Value
DMP-1/Ods	0	5	11.6	4	10.0	9	10.8	*p* > 0.05
	1	7	16.3	12	30.0	19	22.9	*p* > 0.05
	2	31	72.1	24	60.0	55	66.3	*p* > 0.05
DMP-1/D	0	3	7.0	2	5.0	5	6.0	*p* > 0.05
	1	5	11.6	5	12.5	10	12.0	*p* > 0.05
	2	35	81.4	33	82.5	68	81.9	*p* > 0.05
DSPP/Ods	0	3	7.0	4	10.0	7	8.4	*p* > 0.05
	1	6	14.0	7	17.5	13	15.7	*p* > 0.05
	2	34	79.1	29	72.5	63	75.9	*p* > 0.05
DSPP/D	0	32	74.4	31	77.5	63	75.9	*p* > 0.05
	1	4	9.3	4	10.0	8	9.6	*p* > 0.05
	2	7	16.3	5	12.5	12	14.5	*p* > 0.05
OPN/Ods	0	2	4.7	5	12.5	7	8.4	*p* > 0.05
	1	2	4.7	7	17.5	9	10.8	*p* > 0.05
	2	39	90.7	28	70.0	67	80.7	*p* = 0.020
OPN/D	0	12	27.9	15	37.5	27	32.5	*p* > 0.05
	1	4	9.3	5	12.5	9	10.8	*p* > 0.05
	2	27	62.8	20	50.0	47	56.6	*p* > 0.05

Note: Two gender groups: female and male; scoring system (0—no expression, 1—weak, 2—strong expression); Statistical test: χ^2^, significance at *p* < 0.05.

**Table 5:** Comparison of DMP-1, DSPP, and OPN expression in different dentin areas.

Marker	Score	Masticatory n (%)	Frontal n (%)	Total n (%)	*p*-Value
DMP-1/Ods	0	6 (9.4%)	3 (15.8%)	9 (10.8%)	*p* > 0.05
	1	15 (23.4%)	4 (21.1%)	19 22.9%)	*p* > 0.05
	2	43 (67.2%)	12 (63.2%)	55 (66.3%)	*p* > 0.05
DMP-1/D	0	4 (6.3%)	1 (5.3%)	5 (6.0%)	*p* > 0.05
	1	8 (12.5%)	2 (10.5%)	10 (12.0%)	*p* > 0.05
	2	52 (81.3%)	16 (84.2%)	68 (81.9%)	*p* > 0.05
DSPP/Ods	0	5 (7.8%)	2 (10.5%)	7 (8.4%)	*p* > 0.05
	1	9 (14.1%)	4 (21.1%)	13 (15.7%)	*p* > 0.05
	2	50 (78.1%)	13 (68.4%)	63 (75.9%)	*p* > 0.05
DSPP/D	0	51 (79.7%)	12 (63.2%)	63 (75.9%)	*p* > 0.05
	1	5 (7.8%)	3 (15.8%)	8 (9.6%)	*p* > 0.05
	2	8 (12.5%)	4 (21.1%)	12 (14.5%)	*p* > 0.05
OPN/Ods	0	6 (9.4%)	1 (5.3%)	7 (8.4%)	*p* > 0.05
	1	8 (12.5%)	1 (5.3%)	9 (10.8%)	*p* > 0.05
	2	50 (78.1%)	17 (89.5%)	67 (80.7%)	*p* > 0.05
OPN/D	0	25 (39.1%) (B)	2 (10.5%)	27 (32.5%)	*p* < 0.01
	1	9 (14.1%)	0 (0.0%)	9 (10.8%)	*p* > 0.05
	2	30 (46.9%)	17 (89.5%) (A)	47 (56.6%)	*p* < 0.01

Note: A scoring system was used (0—no expression, 1—weak, 2—strong expression); anatomical regions (masticatory and frontal); using the χ^2^ test; *p* < 0.05. Statistically significant differences were observed only for OPN/D between masticatory and frontal teeth (*p* < 0.01), with frontal teeth showing higher expression (score 2) and lower expression (score 0). All other markers showed no significant differences by tooth type.

**Table 6:** Distribution of biomarker scores in upper and lower teeth.

Marker	Score	Upper Tooth (n, %)	Lower Tooth (n, %)	Total (n, %)	*p*-Value
DMP-1/Ods	0	1 (2.2%)	8 (21.1%)	9 (10.8%)	*p* = 0.006
	1	12 (26.7%)	7 (18.4%)	19 (22.9%)	*p* > 0.05
	2	32 (71.1%)	23 (60.5%)	55 (66.3%)	*p* > 0.05
DMP-1/D	0	0 (0.0%)	5 (13.2%)	5 (6.0%)	*p* = 0.013
	1	6 (13.3%)	4 (10.5%)	10 (12.0%)	*p* > 0.05
	2	39 (86.7%)	29 (76.3%)	68 (81.9%)	*p* > 0.05
DSPP/Ods	0	6 (13.3%)	1 (2.6%)	7 (8.4%)	*p* = 0.12
	1	7 (15.6%)	6 (15.8%)	13 (15.7%)	*p* > 0.05
	2	32 (71.1%)	31 (81.6%)	63 (75.9%)	*p* > 0.05
DSPP/D	0	34 (75.6%)	29 (76.3%)	63 (75.9%)	*p* = 0.78
	1	7 (15.6%)	1 (2.6%)	8 (9.6%)	*p* > 0.05
	2	4 (8.9%)	8 (21.1%)	12 (14.5%)	*p* > 0.05
OPN/Ods	0	2 (4.4%)	5 (13.2%)	7 (8.4%)	*p* = 0.09
	1	4 (8.9%)	5 (13.2%)	9 (10.8%)	*p* > 0.05
	2	39 (86.7%)	28 (73.7%)	67 (80.7%)	*p* > 0.05
OPN/D	0	13 (28.9%)	14 (36.8%)	27 (32.5%)	*p* = 0.18
	1	5 (11.1%)	4 (10.5%)	9 (10.8%)	*p* > 0.05
	2	27 (60.0%)	20 (52.6%)	47 (56.6%)	*p* > 0.05

Note: Data present biomarker expression scores (0—no expression, 1—weak, 2—strong expression) in upper and lower teeth, shown as counts with corresponding percentages (n, %).

## Data Availability

The data presented in this study are available on request from the corresponding author. The data are not publicly available due to privacy and ethical restrictions related to the patient informed consent.
